# A Unique Case of Adoption in Golden Snub-Nosed Monkeys

**DOI:** 10.3390/ani14213075

**Published:** 2024-10-25

**Authors:** Haitao Zhao, Jiaxuan Li, Yan Wang, Nianlong Li, Ruliang Pan, Baoguo Li

**Affiliations:** 1 Shaanxi Key Laboratory for Animal Conservation, Northwest University, Xi’an 710069, China; zht@xab.ac.cn (H.Z.); nianlong@stumail.nwu.edu.cn (N.L.); 2 Shaanxi Provincial Field Observation & Research Station for Golden Monkey, Giant Panda and Biodiversity, Shaanxi Institute of Zoology, Xi’an 710032, China; wy0218@xab.ac.cn; 3 International Centre of Biodiversity and Primate Conservation Centre, Dali University, Dali 671000, China; 4 Graduate School of Management, University of California Davis, Davis, CA 95616, USA; 5 School of Human Sciences, University of Western Australia, Perth, WA 6009, Australia; 6 College of Life Science, Yanan University, Yanan 710032, China

**Keywords:** Old World monkeys, sexual selection, allomaternal care, infanticide, cognitive ability

## Abstract

Adoption among nonhuman primates (hereafter primates) has been widely reported, particularly in chimpanzees, renowned for their higher intelligence and well-developed cognition. In contrast to adoption in other Old World monkeys, this case of adoption in golden snub-nosed monkeys (*Rhinopithecus roxellana*) involves two infants associated with three units characterized by distinct social structures and reproductive functions. Consequently, this case extends beyond traditional hypotheses on allomaternal care and adoption—such as enhancing the fitness of adoptive mothers, fostering maternal behaviors, and improving fitness through social and individual interactions—to necessitate an association with the complex social structure characterized by hierarchical, multilevel composition, akin to human society, and intense sexual selection that frequently results in infanticide. Specifically, adopting an infant with a genetic link to the dominant male of the adopting female may reduce the risk of infanticide against her offspring that do not share a biological link with the dominant male. This adoption pattern suggests that golden snub-nosed monkeys may possess more sophisticated intelligence and cognition, characteristics supported by more developed brain structure and facial muscles than the other Old World monkeys.

## Simple Summary

Adoption in nonhuman primates has been frequently reported—infants no longer receive care from their biological parents or allomaternal support but are being nurtured by females with whom they share no biological relationship. The case reported in this study in golden snub-nosed monkeys (*Rhinopithecus roxellana*) extends beyond traditional hypotheses on allomaternal care, which is considered to be associated with the complex social structure characterized by hierarchical, multilevel composition akin to human society, in which intense sexual selection can frequently result in infanticide. Strategically, adopting an infant with a genetic link to the dominant male of the adopting female may reduce the risk of infanticide against her offspring without a biological link with the dominant male. This adoption pattern suggests that *R. roxellana* may possess more sophisticated intelligence and cognition than the other Old World monkeys, referring to its complicated social structure, more developed brain structure, and facial muscles.

## 1. Introduction

**Figure 1:**
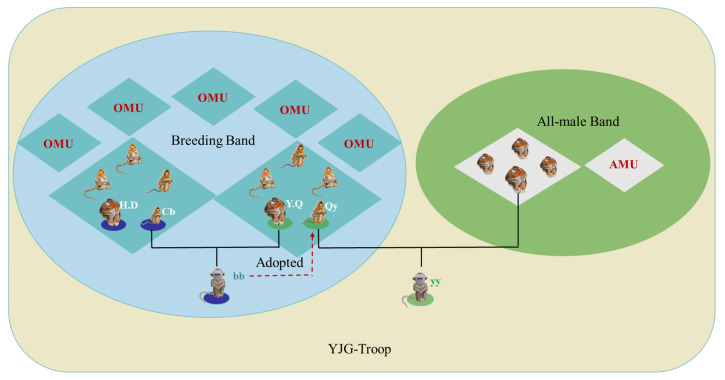
Adult composition of golden snub-nosed monkeys (*Rhinopithecus roxellana*) in OMUs (**left**) and AMU (**right**) involved in the adoption case reported in this study.

This event was recorded in 2020 in a wild golden snub-nosed monkey (*Rhinopithecus roxellana*) troop in the Guanyinshan National Nature Reserve on the southern slopes of the Qinling Mountains, Shaanxi Province, China (please see Section 1 of Supplementary Materials). The troop consisted of 87 individuals, including seven one-male and multi-female units (OMUs) and two all-male units (AMUs). Females typically reproduce biennially, with allomaternal infant care behaviors, also known as aunt care, significantly contributing to the development of the species [1,2]. The specific OMU involved in this adoption, YQ, consisted of the dominant male, four adult females, five juveniles, and three infants (Figure 1). All allomaternal care behaviors were meticulously documented through direct observations and video recordings (please see the Section 2.1 of Supplementary Materials). The adopting female (Qy) had previously given birth to four offspring. Hair samples from all adult individuals and newborn infants in 2020 (43 in total) were analyzed to verify the biological relationships between infants and their parents and determine the adopted infant’s origin and genetic relationships with other group members (please see Sections 2.2 and 4.2 of Supplementary Materials).

## 2. Detailed Case Description

**Figure 2:**
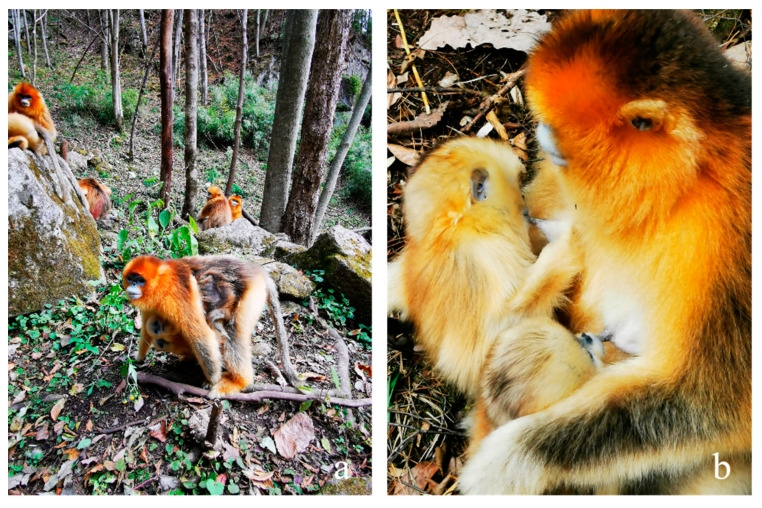
Foster mother (Qy) carrying (**a**) and nursing (**b**) two infants (*bb* and *yy*).

On 3 April 2020, Qy gave birth to a female infant (*yy*), sired by a male from the AMU. Subsequently, on 18 April, Qy was observed at a notable distance from her OMU (YQ), concurrently carrying and nursing two infants, *yy* and *bb*, the latter of whom was less than one month old. Over the following two days, Qy remained within the vicinity of YQ but isolated from the other members, displaying cautious behavior when others approached. Several females from the troop attempted to engage with the infants, exhibiting potential allomaternal interest, but Qy consistently rebuffed their advances. Genetic analyses revealed that *bb* was the progeny of the female Cb who gave birth for the first time and rarely hugged *bb* from the HD group and the dominant male of the YQ group, thereby establishing no genetic relationship between *bb* and Qy (Figure 1). Despite this, Qy continued to care for both infants in the subsequent days (Figure 2). An adult female, identified as Fb from another OMU, approached Qy (within 0.5 m) and the infants on multiple occasions, attempting to interact with *bb*. These attempts elicited distress vocalizations from *bb*, which, in turn, prompted immediate protective responses from Qy. We included 254 instances of nursing and 325 instances of caregiving (carrying, holding, and grooming) during this period (please see Section 3.1 of Supplementary Materials). No other females were observed nursing the infants during the first month of adoption. Although previous studies have documented allomaternal behaviors in this species [1], the frequency and nature of the nursing behaviors exhibited by Qy significantly surpassed those recorded among other adult females within the troop (N = 28, df = 3, *p* = 0.000 < 0.01). During perceived danger or threats to *yy* and *bb*, Qy exhibited a significantly higher frequency of caregiving behaviors than other aunt-females (N = 28, df = 3, *p* = 0.000 < 0.01), reinforcing her status as the de facto foster mother rather than an auxiliary caregiver for *bb*. Furthermore, the frequency of nursing and caregiving provided by Qy to both *yy* and *bb* was comparable (Z = −1.852, *p* = 0.064 > 0.05; Z = −1.183, *p* = 0.237 > 0.05; see Section 4.1 of Supplementary Materials), indicating an equitable level of care and investment to both infants.

## 3. Discussion

Adoption in wild primates is a common phenomenon observed in various species, including great apes, such as chimpanzees (*Pan troglodytes*), bonobos (*Pan paniscus*), and gorillas (*Gorilla gorilla*) [3,4,5]; howler monkeys (*Alouatta guariba*) [6]; bonnet macaques (*Macaca radiata*) [7]; baboons (*Papio cynocephalus*) [8]; marmosets (*Callithrix jacchus*); and capuchin monkeys (*Cebus libidinosus*) [9]. Adoption typically occurs within the same group, although exceptions exist, such as cross-group adoption in chimpanzees [3], bonobos [5], and black-fronted titi monkeys [10]; cross-genera adoption in marmosets and capuchins [9]; and adoptions by siblings in rhesus macaques (*M. mulatta*) [11] and Japanese macaques (*M. fuscata*) [12], as well as by siblings and grandmothers in chimpanzees [4]. Notably, great apes are distinguished by more complex behavior patterns associated with adoption and allomaternal behaviors, likely due to their higher intelligence and significantly developed cognition [4,5,13].

The occurrence of allomaternal behavior and adoption in animals is thought to enhance the fitness of adoptive mothers by fostering maternal behaviors, increasing individual survival, and improving overall fitness through social and individual interactions [14]. These behaviors also demonstrate reciprocal altruism, which varies across different social systems [4,15]. Although normative allomaternal behaviors have been reported in *Rhinopithecus bieti* in a single OMU, a species from the same genus [16], the specific instance of infant adoption observed in *R. roxellana* has not been previously reported in either this species or genus. Notably, although the adoption process (Figure 1) occurred between two OMUs, it also involved a bachelor group (AMU) with different social structures and reproductive functions. Qy extended care to *bb*, an infant fathered by her dominant OMU male with a female from a different OMU, while also caring for her biological infant (*yy*), who shares no genetic link with the dominant male in her group, thereby effectively bridging the social structures of two OMUs and an AMU. Typically, aunt-female care in this species is limited to the first three months after birth and primarily occurs among related females within the same OMU [16].

In contrast, the foster care provided to *bb* by Qy continued for seven months and involved a more complex level of interaction than observed with *R. bieti*. Hence, the foster care displayed by Qy successfully navigated complex inter- and intra-OMU relationships and interactions between OMUs and AMU, presenting a behavioral dynamic seldom recorded among other primates. This phenomenon is likely associated with the unique social structure of *Rhinopithecus*, which parallels the evolution of hierarchical multilevel social structures in humans driven by hunting and gathering practices [17]. *Rhinopithecus* species underwent various evolutionary processes, evolving from independent OMUs and AMUs into expansive, hierarchical multilevel societies as a unique adaptation to the harsh cold climates of the Pleistocene glaciation [18]. This complex social framework suggests the need for exceptional spatial memory, intelligence, and recognition abilities to manage social and individual challenges similar to those humans face [19]. 

The intense sexual selection may also influence the distinctive adoption strategy used by the adopter (Figure 1) in a multilevel society. Within *Rhinopithecus* species, dominant males exhibit exclusionary and infanticidal behaviors towards non-kin individuals to induce the reproductive readiness of females and ensure the perpetuation of their genetic lineage [20]. This behavior intensifies when a new adult male assumes dominance [20,21,22]. In this case, the adopted infant (*bb*) was identified as the progeny of the dominant male of the adopter’s OMU (YQ) and a female (Cb) from another OMU (HD). In contrast, *yy* was identified as the offspring of the adopter and another male from the AMU, showing no genetic link to the dominant male of her group, thereby facing a significant risk of expulsion or harm. The adoption of *bb*, who has a genetic connection to the dominant male, could be interpreted as an affiliative gesture to elicit a protective or empathetic reaction from the dominant male towards her biological progeny (*yy*). This strategic behavior suggests cognitive sophistication compared to other Old World monkeys. Support for such a hypothesis can also be found in anatomical studies. Notably, *Rhinopithecus* species display more evolved brain structures than macaques, showing a closer resemblance to those of apes [23]. Various features, such as the sub-parietal sulcus, collateral fissures, and hippocampus, indicate more advanced cognitive abilities and capacities [24,25]. Moreover, distinct from other Old World monkeys, members of the *Rhinopithecus* genus exhibit a rudimentary development of the risorius (smiling) muscle, a feature fully present in apes and humans [23]. The presence of this muscle supports a broader range of facial expressions, facilitating non-verbal communication and reflecting higher cognitive capacities.

## 4. Conclusions

Consequently, compared to other Old World monkeys, *Rhinopithecus* species may employ sophisticated strategies to enhance social cohesion and maintain stable relationships, requiring significant cognitive efforts.

## Data Availability

The original data records and other requirements are available by contacting the corresponding or first authors.

## Supplementary Materials

The following supporting information can be downloaded at https://www.mdpi.com/article/10.3390/ani14213075/s1: More detailed information of materials, methods, and results [26,27,28,29,30,31,32].
