# Supplementary material for: A Unique Case of Adoption in Golden Snub-Nosed Monkeys
Source: Animals (Basel). 2024 Oct 25;14(21):3075. doi: 10.3390/ani14213075 (PMC11545503; doi:10.3390/ani14213075)
Supplement: Supplementary file 1 [file animals-14-03075-s001.zip › animals-3242185-supplementary.pdf]

animals

MDPI

Supplementary Materials

1. Study site and species

The research site ranges from 1,200 to 2,630 m above sea level. The vertical distribution of vegetation at the site can be classified into mixed deciduous broadleaf-coniferous forest (>2,200 m) and deciduous broadleaf forest (1,200–2,200). Observations and food provisioning were conducted in a 20 m × 25 m area at Erdaohe (1,530) in Yanjiagou Valley within the troop's home range. The troop was herded to the site every morning and provided with apples, radishes, and corn thrice daily at 9:00 am, 12:00 pm, and 3:00 pm. All food was presented randomly to ensure the monkeys could freely move around the site without influencing their natural behavior.

The targeted two troops of golden snub-nosed monkeys (*Rhinopithecus roxellana*) inhabit the Dapingyu region of Guanyinshan National Nature Reserve on the southern slopes of the Qinling Mountains, Shaanxi Province, China (107.98°~107.99°E, 33.66°~33.71°N). We focused on the provisioned troop, which is well-habituated to human presence and can be observed at distances of 5–20 m. It comprised 87 individuals, including 36 adults and 51 non-adults, belonging to seven one-male, multi-female units (OMUs). The researchers could quickly identify all the individuals based on physical characteristics such as facial features and body size [26]. This species exhibits a complex multi-level social structure, with dominant hierarchies among females within OMUs and female migration between OMUs, leading to frequent inter-unit mating [27, 28]. Reproductive patterns are highly seasonal, with mating occurring from September to December and births from March to May [26, 29]. Non-maternal (allomaternal) infant care is commonly observed after birth, enhancing infant survival and maternal fitness, notably, over 87% of infants benefit from allomaternal nursing in the first three months of life [2].

2. Data collection

2.1. Behavioral data

The research was conducted from March 2020 to January 2021. Records on allomaternal behavior were collected from 10:00 am to 12:00 pm and 1:00 pm to 3:00 pm (4 h) daily. Newborn infants recorded in 2020 were considered target individuals. Repeated observations were carried out in April, May, and October-November 2020, over a period of 14 weeks. We used focal animal sampling with all-occurrence recording to document the direction, frequency, and consequence of allomaternal behavior for each target infant in each OMU. Observation times were equal among target OMUs. Allomaternal behaviors were defined as actions by females, excluding the mother, contributing to the care of an infant, including nursing, carrying, holding, grooming, protecting, sitting, and resting in contact with the infant [30].

Adoption was defined as instances where infants no longer received care from their biological parents or allomaternal support but continued to be nurtured for more than three months by females with whom they shared no biological relationship. When adoption was observed, the related infants would be dyed with non-toxic dyes for the first time to identify different individuals visually and then collect their behavioral data and hair samples.

2.2. Hair sample collection for analysis

Five to six tail root hair samples from each monkey, a total of 43 individuals, including all seven infants born in 2020 and all relevant adults, were collected non-invasively. Seven mother-infant dyads were identified, with initial assumptions of mother-infant relationships based on observations of mothers feeding and carrying their newborns. We collected samples from each individual at least twice to ensure genetic data reliability.

3. Data analysis

3.1. Behavioral data

Behavioral data were categorized and analyzed using Microsoft Excel for Windows and SPSS v16.0 (SPSS Inc., Chicago, USA). Statistical analysis focused on allomaternal behavior, including nursing and caregiving behaviors (carrying, holding, and grooming), which were repeatedly measured during observation. The Friedman test was applied to determine differences among adult females in response to newborn infants. The Mann-Whitney U test was used to identify differences in Qy nursing of *yy* and *bb*. Significance was determined using a two-tailed standard with a threshold of $p < 0.05$.

3.2. Parentage analysis

DNA was extracted from hair follicles using an Ezup Column Animal Genomic DNA Purification Kit (B518251, Sangon Biotech, Shanghai, China). Paternity testing for all seven infants born in 2020 was performed using 18 highly polymorphic microsatellite loci (D10s1432, D10s2483, D10s676, D12s375, D14s306, D19s248, D19s582, D21s2054, D3s1766, D18s1371, D6s501, D6s1040, D7s1804, D7s2204, D7s820, D8s1049, D9s252, and D19s1034). Microsatellite loci analysis, polymerase chain reaction (PCR) amplification, and genotyping were conducted as described previously [31].

Simple sequence repeat (SSR) data was analyzed for all infants using GeneMapper 5 software. Paternity was assessed using PARENTAGE [32] based on the 18 microsatellite markers, from which kinship relationships were determined.

4. Results

4.1. Allomaternal behavior

Focusing on behaviors and associations relevant to the two infants (*yy* and *bb*) and Qy, we collected data associated with allomaternal behaviors twice a week, from 10:00 am–12:00 pm and 1:00 pm–3:00 pm, during April, May, October, and

November 2020, spanning a total of 14 weeks. We included 254 instances of nursing and 325 instances of caregiving (carrying, holding, and grooming) during this period. In the initial month of adoption, no other females from the OMUs were observed nursing either infant. The frequency of nursing (235) and caregiving behaviors (19) performed by Qy significantly exceeded that of any other adult female within the troop (237; 88) ($N = 28$, $df = 3$, $P = 0.000 < 0.01$; $N = 28$, $df = 3$, $P = 0.000 < 0.01$), while the frequency of nursing and caregiving behaviors provided to *yy* and *bb* by Qy showed no significant difference ($Z = -1.852$, $P = 0.064 > 0.05$; $Z = -1.183$, $P = 0.237 > 0.05$).

4.2. Parentage

This study analyzed 18 microsatellite loci using PARENTAGE and conducted a simulation parentage analysis. Results identified significant thresholds for parentage inference, with a Logarithm of Odds (LOD) score of 4.404 representing a highly significant critical delta ($\Delta = 0.01$) and a LOD score of 2.298 denoting a significant critical delta ($\Delta = 0.05$). Scores exceeding these thresholds indicated genuine parent-offspring relationships. Specifically, our results confirmed that *yy* was the offspring of Qy (LOD = 6.495; $\Delta < 0.01$) and a male other than the dominant male of the YQ unit (LOD = −0.738; $\Delta > 0.05$), while *bb* was confirmed to be the infant of Cb (HD unit) and the dominant male of the YQ unit (LOD = 3.662; $\Delta < 0.05$ and LOD = 3.879; $\Delta < 0.05$, respectively).
